# Supplementary material for: Identification of Freezing-Responsive microRNAs and Their Targets in Chinese Jujube by Small RNA and Degradome Sequencing
Source: Int J Mol Sci. 2026 Sep 4;27(17):7912. doi: 10.3390/ijms27177912 (PMC13566890; doi:10.3390/ijms27177912)
Supplement: Supplementary file 1 [file ijms-27-07912-s001.zip › Supplementary Material S2/novel_miR_81.pdf]

Provisional ID : novel_miR_81
Score total : 432.7
Score for star read(s) : 3.9
Score for read counts : 425.8
Score for mfe : 1.4
Score for randfold : 1.6
Score for cons. seed :
Total read count : 847
Mature read count : 822
Loop read count : 0
Star read count : 25

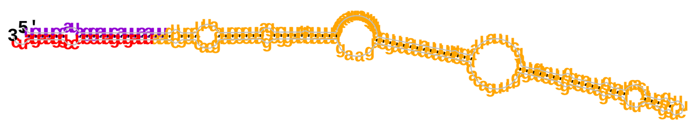

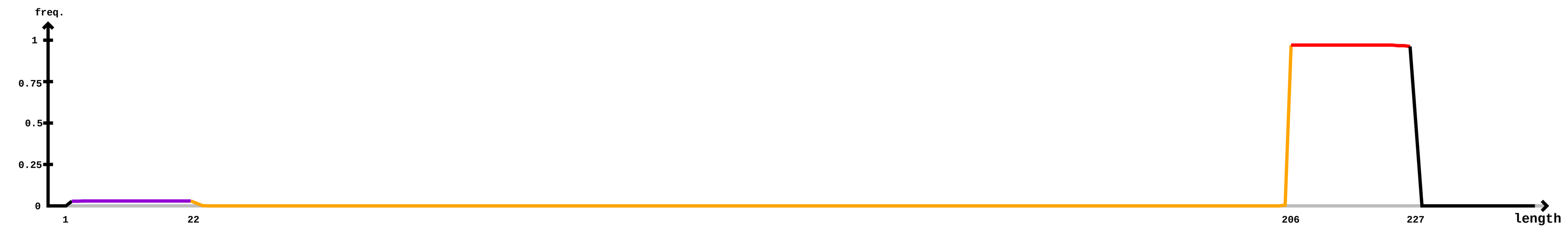

Star Mature

```
5'- gaucuuccauaggaucauuaauuguugauuaaauggguuaggcuuguauuuuggaaaauuuauggguuagguuuauauuuuuggaauuauuucgaguugaguugggauuggauucacuuuggcuucuggcaauuguugaaucuggacuagcguuugacacuccaaaauauaagcugaagaaaauuaggcgaacccaugcacucgcuaauuagugauccuccggaagaucauaauccaguuuggaaccaauu -3' obs
    gaucuuccauaggaucauuaauuguugauuaaauggguuaggcuuguauuuuggaaaauuuauggguuagguuuauauuuuuggaauuauuucgaguugaguugggauuggauucacuuuggcuucuggcaauuguugaaucuggacuagcguuugacacuccaaaauauaagcugaagaaaauuaggcgaacccaugcacucgcuaauuagugauccuccggaagaucauaauccaguuuggaaccaauu     exp
    ((((((((..((((((((((((((.(((...(((((((..(((((.(((((...............(((((((((((.(((.........(((.((((.(((((.(((.....(((.(....).)))..))).))))).)))))))........))))))))))))))....))))))))))).)))))))....))).))))))))))))))..))))))))....((((...)))).......   reads   mm   sample
    ..ucuuccauaggaucauuaauu....................................................................................................................................................................................................................................   1   0   JT-3
    ..ucuuccauaggaucauuaauu....................................................................................................................................................................................................................................   2   0   LT-3
    ..ucuuccauaggaucauuaauu....................................................................................................................................................................................................................................   6   0   JCK-1
    ..ucuuccauaggaucauuaauu....................................................................................................................................................................................................................................   1   0   JT-2
    ..ucuuccauaggaucauuaauu....................................................................................................................................................................................................................................   4   0   JCK-2
    ..ucuuccauaggaucauuaauu....................................................................................................................................................................................................................................   10   0   JCK-3
    ....uuccauaggaucauuaauugu..................................................................................................................................................................................................................................   1   0   JCK-3
    ...............................................................................................................................................................................................................auuagugauccuccggaag.........................   1   0   JCK-2
    ...............................................................................................................................................................................................................Cuuagugauccuccggaagauc......................   2   1   JCK-1
    ...............................................................................................................................................................................................................Cuuagugauccuccggaagauc......................   1   1   JCK-3
    ................................................................................................................................................................................................................uuagugauccuccggaag.........................   1   0   JCK-1
    ................................................................................................................................................................................................................uuagugauccuccggaag.........................   1   0   JCK-2
    ................................................................................................................................................................................................................uuagugauccuccggaagUu.......................   2   1   LT-2
    ................................................................................................................................................................................................................uuagugauccuccggaagau.......................   1   0   JCK-3
    ................................................................................................................................................................................................................uuagugauccuccggaagUu.......................   1   1   JCK-1
    ................................................................................................................................................................................................................uuagugauccuccggaagauc......................   8   0   LCK-3
    ................................................................................................................................................................................................................uNagugauccuccggaagauc......................   1   1   JCK-2
    ................................................................................................................................................................................................................uuagugauccuccggaCgauc......................   1   1   JCK-2
    ................................................................................................................................................................................................................uuagugauccuccggaagauc......................   220   0   JCK-3
    ................................................................................................................................................................................................................uuagugauccuccggaagauU......................   1   1   JCK-2
    ................................................................................................................................................................................................................uuagugauccuccggaagCuc......................   1   1   JCK-1
    ................................................................................................................................................................................................................uuagugauccuccggaagauc......................   8   0   LT-1
    ................................................................................................................................................................................................................uuagugauccuccggaagauc......................   6   0   LT-3
    ................................................................................................................................................................................................................uuagugauccuccggaagauc......................   326   0   JCK-2
    ................................................................................................................................................................................................................uuagugauccuccggaagauU......................   2   1   JCK-3
    ................................................................................................................................................................................................................uuagugauccuccggaagauU......................   1   1   LT-3
    ................................................................................................................................................................................................................uuagugauccuccggaagauc......................   23   0   JT-3
    ................................................................................................................................................................................................................uuagugauccuccggaagauc......................   21   0   JT-2
    ................................................................................................................................................................................................................uuagugaucAuccggaagauc......................   1   1   JCK-2
    ................................................................................................................................................................................................................uuagugauccuccggaagauc......................   177   0   JCK-1
    ................................................................................................................................................................................................................uuCgugauccuccggaagauc......................   1   1   JCK-2
    ................................................................................................................................................................................................................uuagugauUcuccggaagauc......................   1   1   JCK-2
    ................................................................................................................................................................................................................uuCgugauccuccggaagauc......................   1   1   JT-2
```

```
  Star                                                                                                                                                                                                                   Mature
gaucuuccauaggaucauuaauuguugauuaaauggguuaggcuuguauuuuggaaaauuuauggguuagguuuauauuuuuggaauuauuucgaguugaguugggauuggauucacuuuggcuucuggcaauuguugaaucuggacuagcguuugacacuccaaaauauaagcugaagaaaauuaggcgaacccaugcacucgcuaauuagugauccuccggaagaucauaauccaguuuggaaccaauu
................................................................................................................................................................................................................uuagugauccuccggaagauc......................    12    0    LT-2
```
